# 2-(4-Amino­phen­yl)-1-phenyl­diazenium 2,4,6-trinitro­phenolate

**DOI:** 10.1107/S1600536811008968

**Published:** 2011-03-12

**Authors:** Graham Smith, Urs D. Wermuth, Jonathan M. White

**Affiliations:** a Faculty of Science and Technology, Queensland University of Technology, GPO Box 2434, Brisbane, Queensland 4001, Australia; b BIO-21 Molecular Science and Biotechnology, University of Melbourne, Parkville, Victoria 3052, Australia

## Abstract

In the title salt, C_12_H_12_N_3_^+^·C_6_H_2_N_3_O_7_^−^, the diazenyl group of the 4-(phenyl­diazen­yl)aniline mol­ecule is protonated and forms a hydrogen bond with the phenolate O-atom acceptor of the picrate anion. Structure extension occurs through two symmetrical inter-ion three-centre amine N—H⋯O,O′_nitro_ hydrogen-bonding associations [graph set *R*_1_^2^(4)], giving a convoluted two-dimensional network structure.

## Related literature

For the diazo-dye precursor aniline yellow [4-(phenyl­diazen­yl)aniline], see: O’Neil (2001 ▶). For structural data on diazenyl-protonated salts of aniline yellow, see: Yatsenko *et al.* (2000 ▶); Mahmoudkhani & Langer (2001*a* ▶); Smith *et al.* (2009 ▶). For amine-protonated salts of aniline yellow, see: Mahmoudkhani & Langer (2001*b* ▶); Smith *et al.* (2008 ▶). For hydrogen-bonding graph-set analysis, see: Etter *et al.* (1990 ▶).
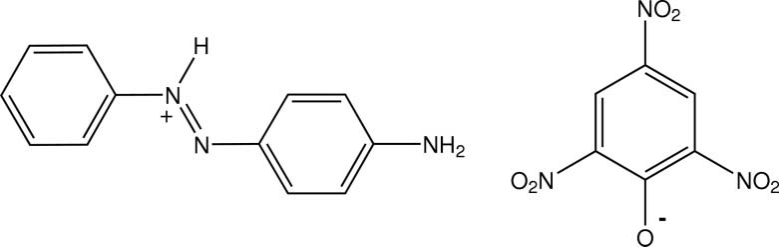


## Experimental

### 

#### Crystal data

C_12_H_12_N_3_^+^·C_6_H_2_N_3_O_7_^−^
*M* *_r_* = 426.35
Monoclinic, 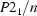

*a* = 5.4506 (2) Å
*b* = 16.8974 (5) Å
*c* = 19.9386 (6) Å
β = 94.063 (3)°
*V* = 1831.75 (10) Å^3^
*Z* = 4
Mo *K*α radiation
μ = 0.12 mm^−1^
*T* = 180 K
0.35 × 0.18 × 0.15 mm

#### Data collection

Oxford Diffraction Gemini-S CCD detector diffractometer
Absorption correction: multi-scan (*CrysAlis PRO*; Oxford Diffraction, 2010 ▶) *T*_min_ = 0.885, *T*_max_ = 0.980
12224 measured reflections
3593 independent reflections
2278 reflections with *I* > 2σ(*I*)
*R*_int_ = 0.035

#### Refinement

*R*[*F* ^2^ > 2σ(*F* ^2^)] = 0.037
*wR*(*F* ^2^) = 0.075
*S* = 0.87
3593 reflections
292 parameters
H atoms treated by a mixture of independent and constrained refinement
Δρ_max_ = 0.14 e Å^−3^
Δρ_min_ = −0.23 e Å^−3^

### 

Data collection: *CrysAlis PRO* (Oxford Diffraction, 2010 ▶); cell refinement: *CrysAlis PRO*; data reduction: *CrysAlis PRO*; program(s) used to solve structure: *SIR92* (Altomare *et al.*, 1994 ▶); program(s) used to refine structure: *SHELXL97* (Sheldrick, 2008 ▶) within *WinGX* (Farrugia, 1999 ▶); molecular graphics: *PLATON* (Spek, 2009 ▶); software used to prepare material for publication: *PLATON*.

## Supplementary Material

Crystal structure: contains datablocks global, I. DOI: 10.1107/S1600536811008968/fl2338sup1.cif

Structure factors: contains datablocks I. DOI: 10.1107/S1600536811008968/fl2338Isup2.hkl

Additional supplementary materials:  crystallographic information; 3D view; checkCIF report

## Figures and Tables

**Table 1:** Hydrogen-bond geometry (Å, °)

*D*—H⋯*A*	*D*—H	H⋯*A*	*D*⋯*A*	*D*—H⋯*A*
N11—H11⋯O1*A*	0.879 (18)	2.045 (18)	2.9039 (18)	165.4 (16)
N4—H41⋯O41*A*^i^	0.89 (2)	2.44 (2)	3.211 (2)	145.0 (16)
N4—H41⋯O42*A*^i^	0.89 (2)	2.29 (2)	3.127 (2)	156.9 (15)
N4—H42⋯O61*A*^ii^	0.88 (2)	2.33 (2)	3.170 (2)	159 (2)
N4—H42⋯O62*A*^ii^	0.88 (2)	2.36 (2)	3.126 (2)	145 (2)

Symmetry codes: (i) 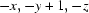; (ii) 
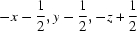
.

**Fig. 1.**
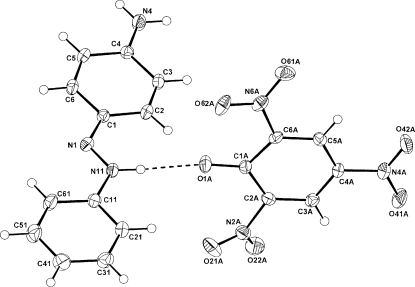
Molecular conformation and atom naming scheme for the diazenyl-protonated cation and the picrate anion in (I). The inter-species hydrogen bond is shown as a dashed line and displacement ellipsoids are drawn at the 40% probability level.

**Fig. 2.**
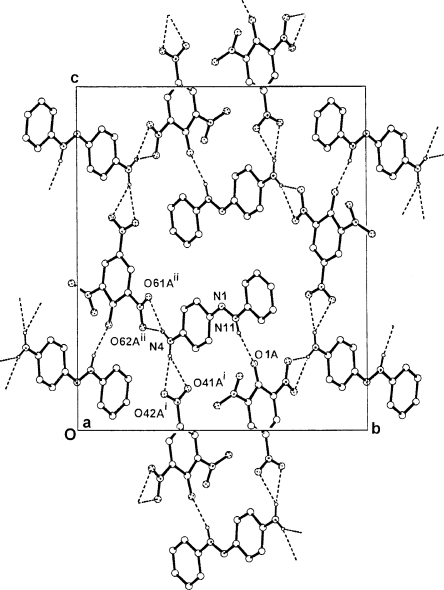
The hydrogen-bonded sheet structure of (I), with non-associative H atoms omitted and hydrogen bonds shown as dashed lines. For symmetry codes, see Table 1.

## supplementary crystallographic information

### Comment

The diazo-dye precursor 4-(phenyldiazenyl)aniline (aniline yellow) (O'Neil, 2001) has been found to react with strong acids to form salts through protonation of the diazenyl group rather than the amine group of the molecule, e.g with the hydrochloride (Yatsenko *et al.*, 2000; Mahmoudkhani & Langer, 2001*a*), and with 5-sulfosalicylic acid (Smith *et al.*, 2009). With benzenesulfonic acid (Smith *et al.*, 2009), the structure of the dichroic salt showed the 1:1 presence of both the diazenyl- and the amine-protonated forms. The phenylhydrazin-1-ium salts are invariably coloured purple-black or red-black as distinct from the amine-protonated salts which are orange-red *e.g.* the oxalate (Mahmoudkhani & Langer, 2001*b*) and the nitro-substituted phthalates and isophthalates (Smith *et al.*, 2008). Our 1:1 stoichiometric reaction of aniline yellow with picric acid in 80% ethanol-water gave red-black crystals of the title salt, (I), and the structure is reported here.

In the structure of (I) (Fig. 1) the diazenyl group of the 4-(phenyldiazenyl)aniline molecule is protonated and forms a hydrogen bond with the phenolate O acceptor of the picrate anion (Table 1). A secondary weak C2—H2···O1A interaction [3.344 (2) Å] is also present. Structure extension occurs through two symmetrical inter-ion three-centre amine *N*—*H*···*O*,*O*'_nitro_ hydrogen-bonding associations [graph set *R*^2^_1_(4) (Etter *et al.*, 1990)], giving a convoluted two-dimensional network structure (Fig. 2). There are no π–π interactions involving the phenyl rings of the cations [minimum inter-ring centroid separation, 4.058 (1) Å]. In the crystal packing there are three close non-bonding intermolecular interactions associated with the nitro groups: O21A···N6A^iii^, 2.8640 (18) Å and O21A···C6A^iii^, 2.974 (2) Å (symmetry code (iii) *x* + 1, *y*, *z* )  and O22A···N4A^iv^, 2.8987 (19) Å (symmetry code (iv) -*x* + 1, -*y* + 1, -*z* ).

The cation in (I) is essentially planar, the C6—C1—N1—N11 and C21—C11—N11—N1 torsion angles being -174.89 (14) and 176.74 (14)° respectively. With the picrate anion, the two *ortho*-related nitro groups are rotated out of the benzene plane [torsion angles C1A—C2A—N2A—O22A, 145.46 (15)° and C5A—C6A—N6A—O62A, -163.83 (15)°] while the *para*-related nitro group is essentially coplanar with the ring [C3A—C4A—N4A—O42A, 179.40 (15)°].

### Experimental

The title compound was synthesized by heating together under reflux for 10 minutes, 1 mmol quantities of 4-(phenyldiazenyl)aniline (aniline yellow) and picric acid in 50 ml of 80% ethanol-water. After concentration to *ca* 30 ml, partial room temperature evaporation of the hot-filtered solution gave red-black prisms of (I) (m.p. 443–445 K) from which a specimen was cleaved for the X-ray analysis.

### Refinement

Hydrogen atoms involved in hydrogen-bonding interactions were located by difference methods and their positional and isotropic displacement parameters were refined. Other H-atoms were included in the refinement at calculated positions and using a riding-model approximation [C—H = 0.93 Å], with *U*_iso_(H) = 1.2*U*_eq_(C).

### Crystal data

**Table:**

C_12_H_12_N_3_^+^·C_6_H_2_N_3_O_7_^−^	*F*(000) = 880
*M**_r_* = 426.35	*D*_x_ = 1.546 Mg m^−^^3^
Monoclinic, *P*2_1_/*n*	Melting point = 443–445 K
Hall symbol: -P 2yn	Mo *K*α radiation, λ = 0.71073 Å
*a* = 5.4506 (2) Å	Cell parameters from 3903 reflections
*b* = 16.8974 (5) Å	θ = 3.2–28.7°
*c* = 19.9386 (6) Å	µ = 0.12 mm^−^^1^
β = 94.063 (3)°	*T* = 180 K
*V* = 1831.75 (10) Å^3^	Prism, red-black
*Z* = 4	0.35 × 0.18 × 0.15 mm

### Data collection

**Table:**

Oxford Diffraction Gemini-S CCD detector diffractometer	3593 independent reflections
Radiation source: fine-focus sealed tube	2278 reflections with *I* > 2σ(*I*)
graphite	*R*_int_ = 0.035
Detector resolution: 16.077 pixels mm^-1^	θ_max_ = 26.0°, θ_min_ = 3.2°
ω scans	*h* = −6→6
Absorption correction: multi-scan (*CrysAlis PRO*; Oxford Diffraction, 2010)	*k* = −20→20
*T*_min_ = 0.885, *T*_max_ = 0.980	*l* = −22→24
12224 measured reflections	

### Refinement

**Table:**

Refinement on *F*^2^	Primary atom site location: structure-invariant direct methods
Least-squares matrix: full	Secondary atom site location: difference Fourier map
*R*[*F*^2^ > 2σ(*F*^2^)] = 0.037	Hydrogen site location: inferred from neighbouring sites
*wR*(*F*^2^) = 0.075	H atoms treated by a mixture of independent and constrained refinement
*S* = 0.87	*w* = 1/[σ^2^(*F*_o_^2^) + (0.0376*P*)^2^] where *P* = (*F*_o_^2^ + 2*F*_c_^2^)/3
3593 reflections	(Δ/σ)_max_ < 0.001
292 parameters	Δρ_max_ = 0.14 e Å^−^^3^
0 restraints	Δρ_min_ = −0.23 e Å^−^^3^

### Special details

**Table:**

Geometry. Bond distances, angles *etc*. have been calculated using the rounded fractional coordinates. All su's are estimated from the variances of the (full) variance-covariance matrix. The cell e.s.d.'s are taken into account in the estimation of distances, angles and torsion angles
Refinement. Refinement of *F*^2^ against ALL reflections. The weighted *R*-factor *wR* and goodness of fit *S* are based on *F*^2^, conventional *R*-factors *R* are based on *F*, with *F* set to zero for negative *F*^2^. The threshold expression of *F*^2^ > σ(*F*^2^) is used only for calculating *R*-factors(gt) *etc*. and is not relevant to the choice of reflections for refinement. *R*-factors based on *F*^2^ are statistically about twice as large as those based on *F*, and *R*- factors based on ALL data will be even larger.

### Fractional atomic coordinates and isotropic or equivalent isotropic displacement parameters (Å^2^)

**Table:**

	*x*	*y*	*z*	*U*_iso_*/*U*_eq_	
N1	0.5298 (2)	0.49802 (8)	0.36091 (6)	0.0251 (4)	
N4	−0.2497 (3)	0.32118 (10)	0.25985 (9)	0.0344 (6)	
N11	0.6596 (2)	0.54559 (8)	0.32635 (7)	0.0252 (5)	
C1	0.3432 (3)	0.45678 (9)	0.33086 (8)	0.0222 (5)	
C2	0.2730 (3)	0.45249 (10)	0.26070 (8)	0.0269 (6)	
C3	0.0809 (3)	0.40706 (10)	0.23773 (8)	0.0276 (5)	
C4	−0.0594 (3)	0.36418 (9)	0.28318 (8)	0.0238 (5)	
C5	0.0074 (3)	0.36874 (9)	0.35319 (8)	0.0254 (5)	
C6	0.2047 (3)	0.41246 (10)	0.37563 (8)	0.0264 (5)	
C11	0.8581 (3)	0.58730 (10)	0.35919 (8)	0.0242 (5)	
C21	1.0003 (3)	0.63434 (10)	0.32020 (9)	0.0296 (6)	
C31	1.1992 (3)	0.67474 (10)	0.35069 (9)	0.0343 (6)	
C41	1.2563 (3)	0.66787 (11)	0.41880 (9)	0.0385 (7)	
C51	1.1132 (3)	0.62021 (12)	0.45732 (9)	0.0410 (7)	
C61	0.9138 (3)	0.57997 (11)	0.42795 (9)	0.0336 (6)	
O1A	0.5381 (2)	0.60880 (7)	0.19268 (6)	0.0355 (4)	
O21A	0.9876 (2)	0.55846 (7)	0.15684 (7)	0.0441 (5)	
O22A	0.9183 (2)	0.48046 (7)	0.07170 (7)	0.0434 (5)	
O41A	0.5231 (2)	0.62072 (8)	−0.12058 (6)	0.0504 (5)	
O42A	0.2254 (2)	0.70145 (8)	−0.10480 (6)	0.0402 (4)	
O61A	−0.0343 (2)	0.74572 (7)	0.11123 (7)	0.0408 (5)	
O62A	0.2079 (2)	0.72543 (7)	0.20024 (6)	0.0392 (5)	
N2A	0.8675 (2)	0.53839 (9)	0.10537 (8)	0.0300 (5)	
N4A	0.3933 (3)	0.65722 (9)	−0.08305 (7)	0.0330 (5)	
N6A	0.1562 (3)	0.71719 (8)	0.13953 (7)	0.0299 (5)	
C1A	0.5121 (3)	0.62347 (9)	0.13155 (8)	0.0239 (6)	
C2A	0.6597 (3)	0.58734 (9)	0.08148 (8)	0.0238 (5)	
C3A	0.6232 (3)	0.59705 (9)	0.01357 (8)	0.0250 (6)	
C4A	0.4368 (3)	0.64710 (10)	−0.01148 (8)	0.0251 (5)	
C5A	0.2883 (3)	0.68524 (10)	0.03106 (8)	0.0245 (5)	
C6A	0.3236 (3)	0.67494 (9)	0.09927 (8)	0.0231 (5)	
H2	0.36010	0.48120	0.23050	0.0320*	
H3	0.03980	0.40380	0.19170	0.0330*	
H5	−0.08410	0.34180	0.38350	0.0300*	
H6	0.25080	0.41350	0.42140	0.0320*	
H11	0.626 (3)	0.5560 (10)	0.2835 (9)	0.041 (6)*	
H21	0.96250	0.63870	0.27410	0.0360*	
H31	1.29490	0.70670	0.32500	0.0410*	
H41	−0.289 (3)	0.3182 (12)	0.2157 (11)	0.061 (7)*	
H42	−0.337 (4)	0.2946 (13)	0.2877 (12)	0.077 (8)*	
H43	1.39030	0.69510	0.43900	0.0460*	
H51	1.15240	0.61550	0.50330	0.0490*	
H61	0.81770	0.54830	0.45380	0.0400*	
H3A	0.72160	0.57060	−0.01540	0.0300*	
H5A	0.16310	0.71820	0.01350	0.0290*	

### Atomic displacement parameters (Å^2^)

**Table:**

	*U*^11^	*U*^22^	*U*^33^	*U*^12^	*U*^13^	*U*^23^
N1	0.0268 (7)	0.0258 (8)	0.0234 (8)	0.0003 (7)	0.0058 (6)	0.0002 (6)
N4	0.0355 (9)	0.0423 (10)	0.0250 (10)	−0.0121 (8)	−0.0001 (8)	0.0059 (8)
N11	0.0281 (8)	0.0296 (8)	0.0178 (8)	−0.0013 (7)	0.0017 (6)	0.0019 (7)
C1	0.0235 (8)	0.0239 (9)	0.0195 (9)	0.0015 (7)	0.0027 (7)	−0.0006 (7)
C2	0.0299 (9)	0.0301 (10)	0.0212 (10)	−0.0019 (8)	0.0050 (7)	0.0056 (8)
C3	0.0306 (9)	0.0336 (10)	0.0183 (9)	−0.0029 (8)	0.0005 (7)	0.0039 (8)
C4	0.0228 (8)	0.0245 (9)	0.0243 (9)	0.0020 (8)	0.0031 (7)	0.0016 (7)
C5	0.0304 (9)	0.0261 (9)	0.0206 (9)	−0.0019 (8)	0.0080 (7)	0.0029 (7)
C6	0.0333 (9)	0.0287 (10)	0.0174 (9)	0.0033 (8)	0.0041 (7)	0.0003 (7)
C11	0.0252 (9)	0.0253 (9)	0.0222 (9)	0.0004 (8)	0.0020 (7)	−0.0028 (8)
C21	0.0343 (10)	0.0315 (10)	0.0230 (10)	0.0004 (8)	0.0018 (8)	0.0018 (8)
C31	0.0343 (10)	0.0343 (11)	0.0349 (11)	−0.0081 (9)	0.0059 (9)	0.0008 (9)
C41	0.0359 (10)	0.0438 (12)	0.0352 (12)	−0.0103 (9)	−0.0008 (9)	−0.0113 (9)
C51	0.0437 (11)	0.0564 (13)	0.0228 (10)	−0.0103 (10)	0.0018 (9)	−0.0077 (9)
C61	0.0374 (10)	0.0416 (11)	0.0224 (10)	−0.0081 (9)	0.0073 (8)	−0.0027 (9)
O1A	0.0393 (7)	0.0468 (8)	0.0200 (7)	−0.0047 (6)	−0.0011 (5)	0.0078 (6)
O21A	0.0375 (7)	0.0409 (8)	0.0506 (9)	−0.0030 (6)	−0.0194 (7)	0.0055 (7)
O22A	0.0449 (8)	0.0397 (8)	0.0463 (9)	0.0160 (7)	0.0083 (7)	−0.0007 (7)
O41A	0.0556 (8)	0.0749 (10)	0.0222 (7)	0.0169 (8)	0.0133 (6)	−0.0041 (7)
O42A	0.0471 (8)	0.0470 (8)	0.0252 (7)	0.0101 (7)	−0.0069 (6)	0.0066 (6)
O61A	0.0296 (7)	0.0452 (8)	0.0484 (9)	0.0091 (6)	0.0075 (6)	−0.0098 (7)
O62A	0.0582 (8)	0.0378 (8)	0.0236 (8)	−0.0076 (6)	0.0163 (6)	−0.0070 (6)
N2A	0.0259 (8)	0.0291 (9)	0.0350 (9)	−0.0019 (7)	0.0013 (7)	0.0069 (7)
N4A	0.0368 (8)	0.0425 (10)	0.0200 (8)	0.0007 (8)	0.0040 (7)	0.0005 (7)
N6A	0.0347 (9)	0.0266 (8)	0.0297 (9)	−0.0066 (7)	0.0119 (7)	−0.0060 (7)
C1A	0.0241 (9)	0.0270 (10)	0.0204 (10)	−0.0082 (7)	0.0000 (7)	0.0026 (7)
C2A	0.0201 (8)	0.0233 (9)	0.0276 (10)	−0.0005 (7)	−0.0006 (7)	0.0025 (8)
C3A	0.0244 (9)	0.0279 (10)	0.0231 (10)	0.0000 (8)	0.0049 (7)	−0.0041 (7)
C4A	0.0278 (9)	0.0308 (10)	0.0169 (9)	0.0006 (8)	0.0025 (7)	0.0009 (7)
C5A	0.0236 (8)	0.0256 (9)	0.0242 (9)	0.0035 (8)	0.0004 (7)	0.0016 (7)
C6A	0.0231 (9)	0.0252 (9)	0.0216 (9)	−0.0021 (7)	0.0067 (7)	−0.0040 (7)

### Geometric parameters (Å, °)

**Table:**

O1A—C1A	1.242 (2)	C11—C21	1.387 (2)
O21A—N2A	1.225 (2)	C11—C61	1.389 (2)
O22A—N2A	1.230 (2)	C21—C31	1.385 (2)
O41A—N4A	1.2312 (19)	C31—C41	1.377 (3)
O42A—N4A	1.236 (2)	C41—C51	1.390 (3)
O61A—N6A	1.2434 (19)	C51—C61	1.377 (2)
O62A—N6A	1.2311 (18)	C2—H2	0.9300
N1—C1	1.339 (2)	C3—H3	0.9300
N1—N11	1.3002 (18)	C5—H5	0.9300
N4—C4	1.324 (2)	C6—H6	0.9300
N11—C11	1.413 (2)	C21—H21	0.9300
N4—H41	0.89 (2)	C31—H31	0.9300
N4—H42	0.88 (2)	C41—H43	0.9300
N11—H11	0.879 (18)	C51—H51	0.9300
N2A—C2A	1.455 (2)	C61—H61	0.9300
N4A—C4A	1.440 (2)	C1A—C2A	1.460 (2)
N6A—C6A	1.446 (2)	C1A—C6A	1.460 (2)
C1—C6	1.422 (2)	C2A—C3A	1.365 (2)
C1—C2	1.426 (2)	C3A—C4A	1.388 (2)
C2—C3	1.352 (2)	C4A—C5A	1.374 (2)
C3—C4	1.425 (2)	C5A—C6A	1.371 (2)
C4—C5	1.420 (2)	C3A—H3A	0.9300
C5—C6	1.354 (2)	C5A—H5A	0.9300
			
N11—N1—C1	120.61 (13)	C3—C2—H2	120.00
N1—N11—C11	119.37 (13)	C2—C3—H3	120.00
C4—N4—H41	120.3 (12)	C4—C3—H3	120.00
C4—N4—H42	120.3 (15)	C6—C5—H5	120.00
H41—N4—H42	119.4 (19)	C4—C5—H5	120.00
C11—N11—H11	116.8 (11)	C1—C6—H6	119.00
N1—N11—H11	123.7 (11)	C5—C6—H6	119.00
O21A—N2A—O22A	123.37 (13)	C11—C21—H21	120.00
O21A—N2A—C2A	118.30 (14)	C31—C21—H21	120.00
O22A—N2A—C2A	118.29 (14)	C41—C31—H31	120.00
O42A—N4A—C4A	119.03 (14)	C21—C31—H31	120.00
O41A—N4A—C4A	118.78 (14)	C51—C41—H43	120.00
O41A—N4A—O42A	122.19 (14)	C31—C41—H43	120.00
O61A—N6A—C6A	118.63 (14)	C41—C51—H51	120.00
O61A—N6A—O62A	121.87 (15)	C61—C51—H51	120.00
O62A—N6A—C6A	119.49 (14)	C51—C61—H61	120.00
N1—C1—C6	114.41 (14)	C11—C61—H61	120.00
N1—C1—C2	127.45 (15)	O1A—C1A—C2A	123.89 (14)
C2—C1—C6	118.13 (14)	O1A—C1A—C6A	125.39 (15)
C1—C2—C3	120.59 (15)	C2A—C1A—C6A	110.63 (14)
C2—C3—C4	120.74 (15)	N2A—C2A—C1A	117.89 (14)
C3—C4—C5	119.09 (14)	N2A—C2A—C3A	116.65 (14)
N4—C4—C5	121.03 (15)	C1A—C2A—C3A	125.44 (15)
N4—C4—C3	119.89 (15)	C2A—C3A—C4A	118.82 (15)
C4—C5—C6	119.79 (15)	N4A—C4A—C3A	119.61 (15)
C1—C6—C5	121.61 (15)	N4A—C4A—C5A	119.56 (15)
N11—C11—C21	117.80 (14)	C3A—C4A—C5A	120.81 (15)
C21—C11—C61	120.86 (16)	C4A—C5A—C6A	120.36 (15)
N11—C11—C61	121.32 (15)	N6A—C6A—C1A	120.09 (14)
C11—C21—C31	119.20 (16)	N6A—C6A—C5A	116.00 (14)
C21—C31—C41	120.45 (16)	C1A—C6A—C5A	123.90 (15)
C31—C41—C51	119.84 (16)	C2A—C3A—H3A	121.00
C41—C51—C61	120.53 (17)	C4A—C3A—H3A	121.00
C11—C61—C51	119.12 (16)	C4A—C5A—H5A	120.00
C1—C2—H2	120.00	C6A—C5A—H5A	120.00
			
C1—N1—N11—C11	−178.90 (14)	C4—C5—C6—C1	2.4 (2)
N11—N1—C1—C2	5.7 (2)	N11—C11—C21—C31	−178.72 (15)
N11—N1—C1—C6	−174.89 (14)	C61—C11—C21—C31	−0.4 (3)
N1—N11—C11—C21	176.74 (14)	N11—C11—C61—C51	178.27 (16)
N1—N11—C11—C61	−1.6 (2)	C21—C11—C61—C51	0.0 (3)
O21A—N2A—C2A—C1A	−36.9 (2)	C11—C21—C31—C41	0.5 (3)
O21A—N2A—C2A—C3A	141.62 (15)	C21—C31—C41—C51	−0.1 (3)
O22A—N2A—C2A—C1A	145.46 (15)	C31—C41—C51—C61	−0.3 (3)
O22A—N2A—C2A—C3A	−36.1 (2)	C41—C51—C61—C11	0.3 (3)
O41A—N4A—C4A—C5A	−177.77 (16)	O1A—C1A—C2A—N2A	−7.4 (2)
O42A—N4A—C4A—C3A	179.40 (15)	O1A—C1A—C2A—C3A	174.32 (16)
O41A—N4A—C4A—C3A	0.2 (2)	C6A—C1A—C2A—N2A	175.85 (13)
O42A—N4A—C4A—C5A	1.4 (2)	C6A—C1A—C2A—C3A	−2.5 (2)
O61A—N6A—C6A—C1A	−163.87 (14)	O1A—C1A—C6A—N6A	3.5 (2)
O61A—N6A—C6A—C5A	14.6 (2)	O1A—C1A—C6A—C5A	−174.90 (16)
O62A—N6A—C6A—C5A	−163.83 (15)	C2A—C1A—C6A—N6A	−179.79 (14)
O62A—N6A—C6A—C1A	17.7 (2)	C2A—C1A—C6A—C5A	1.8 (2)
C6—C1—C2—C3	−0.4 (2)	N2A—C2A—C3A—C4A	−176.08 (14)
N1—C1—C6—C5	178.82 (15)	C1A—C2A—C3A—C4A	2.3 (2)
N1—C1—C2—C3	179.05 (16)	C2A—C3A—C4A—N4A	−179.13 (15)
C2—C1—C6—C5	−1.7 (2)	C2A—C3A—C4A—C5A	−1.2 (2)
C1—C2—C3—C4	1.7 (3)	N4A—C4A—C5A—C6A	178.58 (15)
C2—C3—C4—N4	178.74 (16)	C3A—C4A—C5A—C6A	0.6 (3)
C2—C3—C4—C5	−1.0 (2)	C4A—C5A—C6A—N6A	−179.50 (15)
C3—C4—C5—C6	−1.1 (2)	C4A—C5A—C6A—C1A	−1.1 (3)
N4—C4—C5—C6	179.24 (16)		

### Hydrogen-bond geometry (Å, °)

**Table:**

*D*—H···*A*	*D*—H	H···*A*	*D*···*A*	*D*—H···*A*
N11—H11···O1A	0.879 (18)	2.045 (18)	2.9039 (18)	165.4 (16)
N4—H41···O41A^i^	0.89 (2)	2.44 (2)	3.211 (2)	145.0 (16)
N4—H41···O42A^i^	0.89 (2)	2.29 (2)	3.127 (2)	156.9 (15)
N4—H42···O61A^ii^	0.88 (2)	2.33 (2)	3.170 (2)	159 (2)
N4—H42···O62A^ii^	0.88 (2)	2.36 (2)	3.126 (2)	145 (2)
C2—H2···O1A	0.93	2.50	3.344 (2)	151
C3A—H3A···O22A^iii^	0.93	2.48	3.384 (2)	163
C5A—H5A···O61A	0.93	2.34	2.663 (2)	100
C21—H21···O62A^iv^	0.93	2.53	3.123 (2)	122
C31—H31···O62A^iv^	0.93	2.52	3.123 (2)	123

Symmetry codes: (i) −*x*, −*y*+1, −*z*; (ii) −*x*−1/2, *y*−1/2, −*z*+1/2; (iii) −*x*+2, −*y*+1, −*z*; (iv) *x*+1, *y*, *z*.

## References

[bb1] Altomare, A., Cascarano, G., Giacovazzo, C., Guagliardi, A., Burla, M. C., Polidori, G. & Camalli, M. (1994). *J. Appl. Cryst.* **27**, 435.

[bb2] Etter, M. C., MacDonald, J. C. & Bernstein, J. (1990). *Acta Cryst.* B**46**, 256–262. 10.1107/s0108768189012929 2344397

[bb3] Farrugia, L. J. (1999). *J. Appl. Cryst.* **32**, 837–838.

[bb4] Mahmoudkhani, A. H. & Langer, V. (2001*a*). *Acta Cryst.* E**57**, o839–o841.

[bb5] Mahmoudkhani, A. H. & Langer, V. (2001*b*). *Acta Cryst.* E**57**, o898–o900.

[bb6] O’Neil, M. J. (2001). Editor. *The Merck Index*, 13th ed., p. 74. Whitehouse Station, New Jersey: Merck & Co.

[bb7] Oxford Diffraction (2010). *CrysAlis PRO* Oxford Diffraction Ltd, Yarnton, England.

[bb8] Sheldrick, G. M. (2008). *Acta Cryst.* A**64**, 112–122. 10.1107/S0108767307043930 18156677

[bb9] Smith, G., Wermuth, U. D., Young, D. J. & White, J. M. (2008). *Acta Cryst.* C**64**, o123–o127. 10.1107/S0108270108001595 18322335

[bb10] Smith, G., Wermuth, U. D., Young, D. J. & White, J. M. (2009). *Acta Cryst.* C**65**, o543–o548. 10.1107/S0108270109036622 19805891

[bb11] Spek, A. L. (2009). *Acta Cryst.* D**65**, 148–155. 10.1107/S090744490804362X PMC2631630 19171970

[bb12] Yatsenko, A. V., Chernyshev, V. V., Kurbakov, A. I. & Schenk, H. (2000). *Acta Cryst.* C**56**, 892–894. 10.1107/s0108270100005357 10935120

